# Supplementary figures and images for: Sexual dimorphism in the Arachnid orders
Source: PeerJ. 2018 Nov 6;6:e5751. doi: 10.7717/peerj.5751 (PMC6225839; doi:10.7717/peerj.5751)

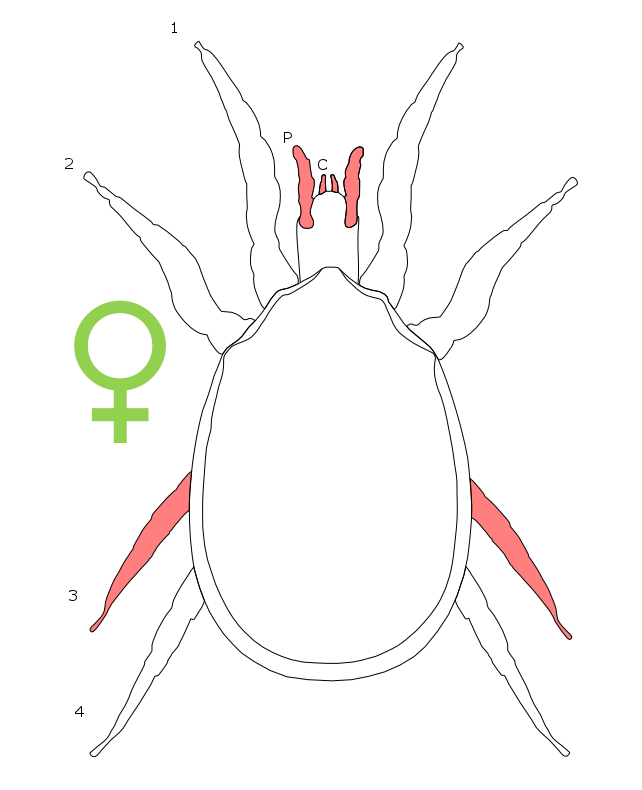

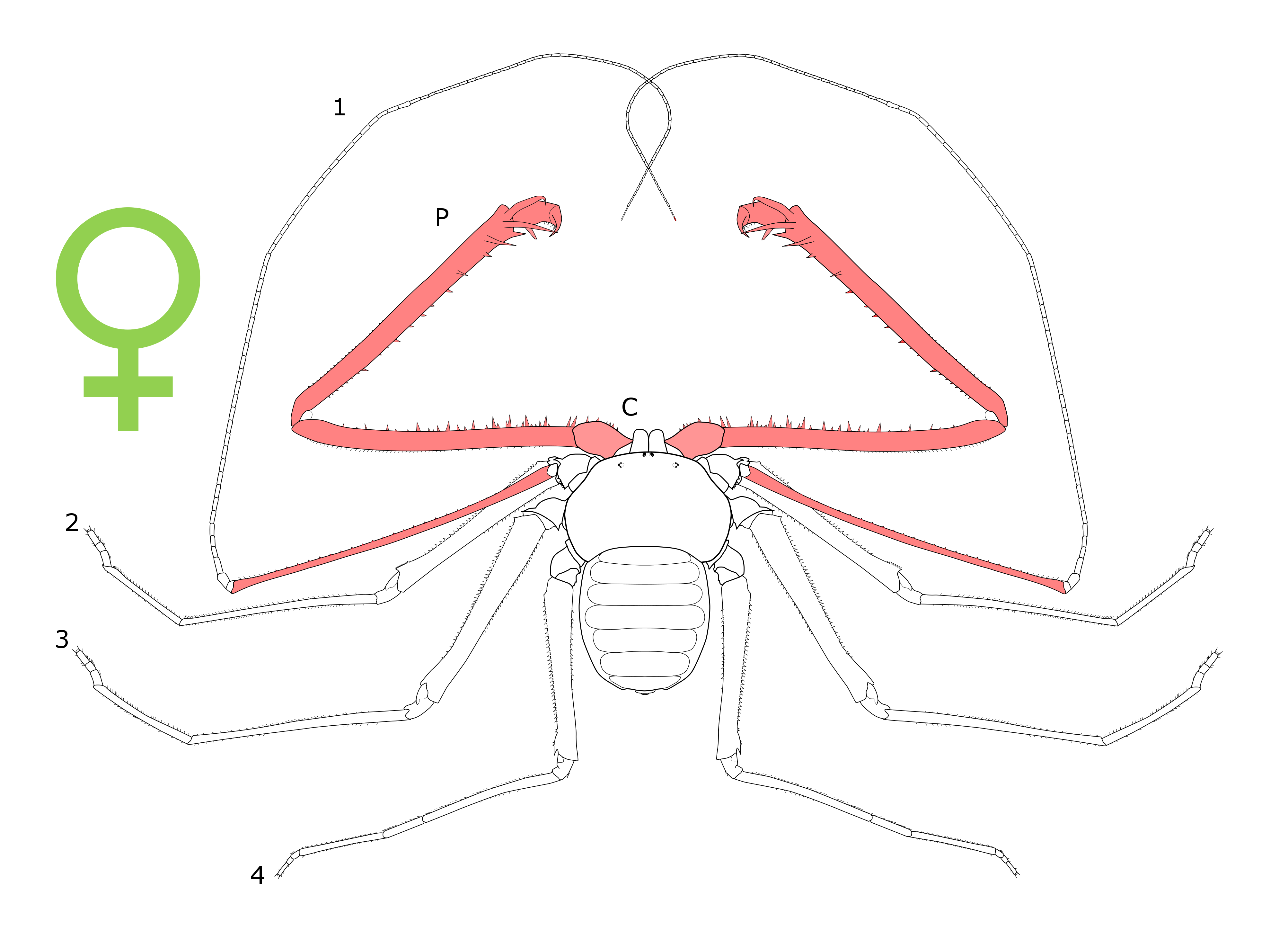

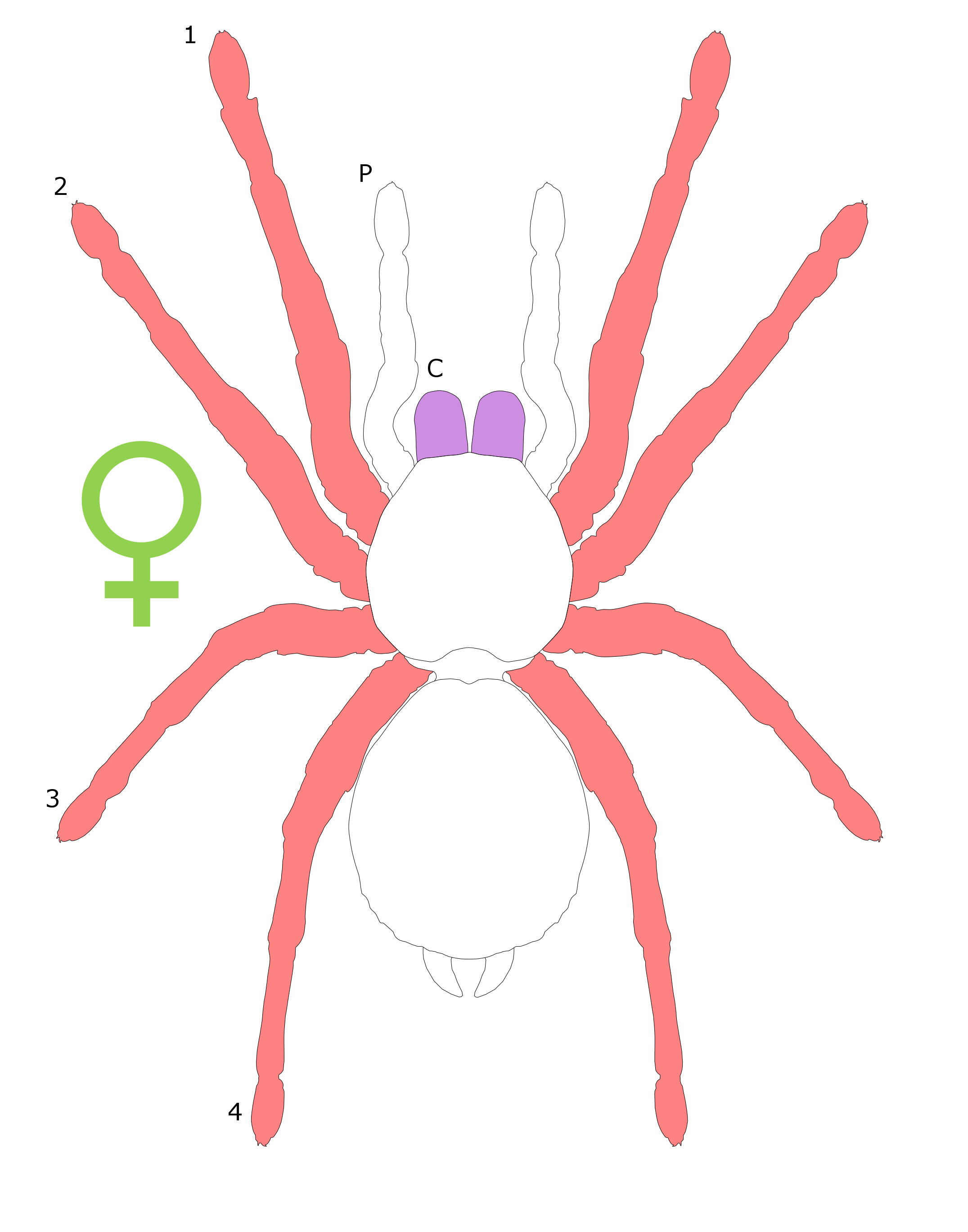

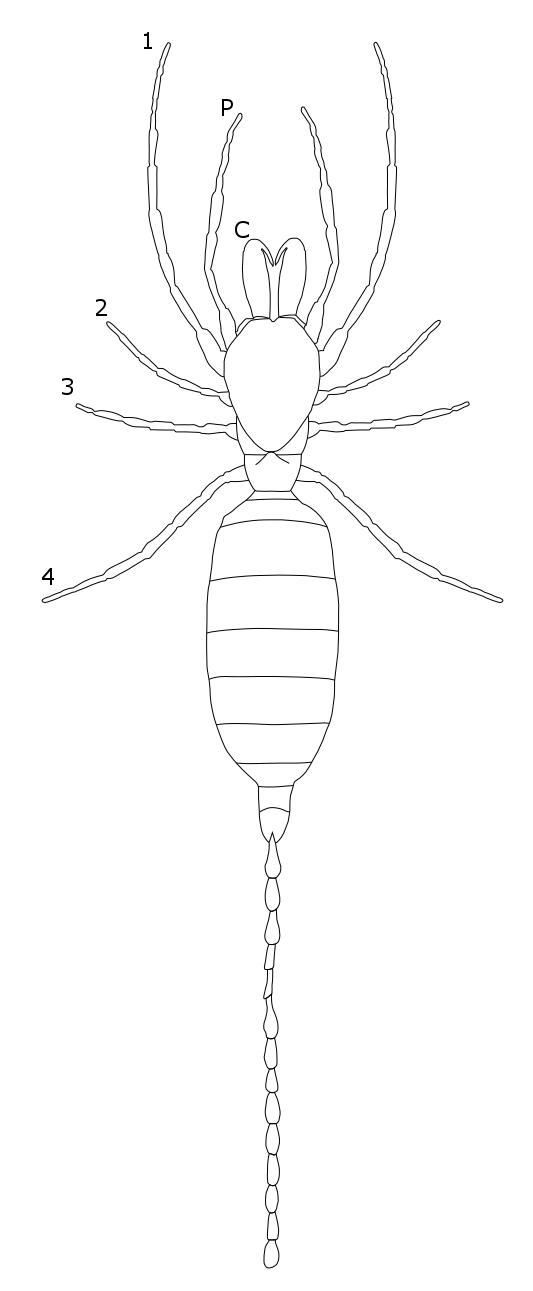

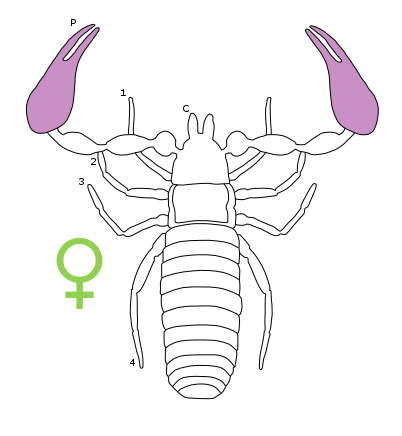

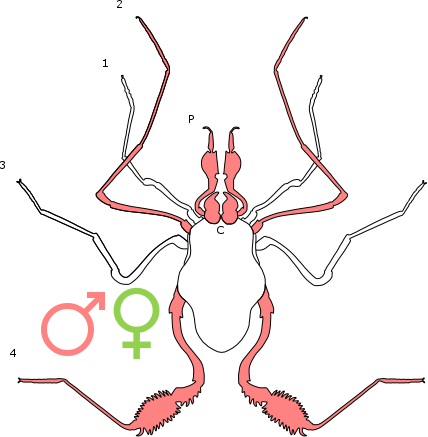

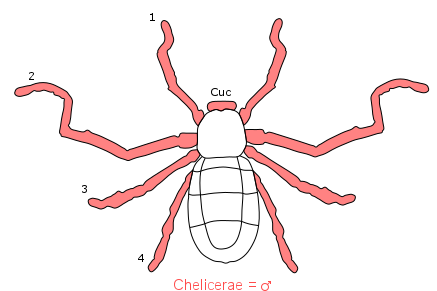

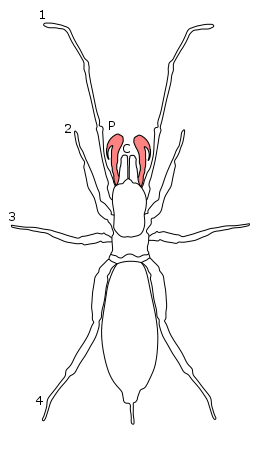

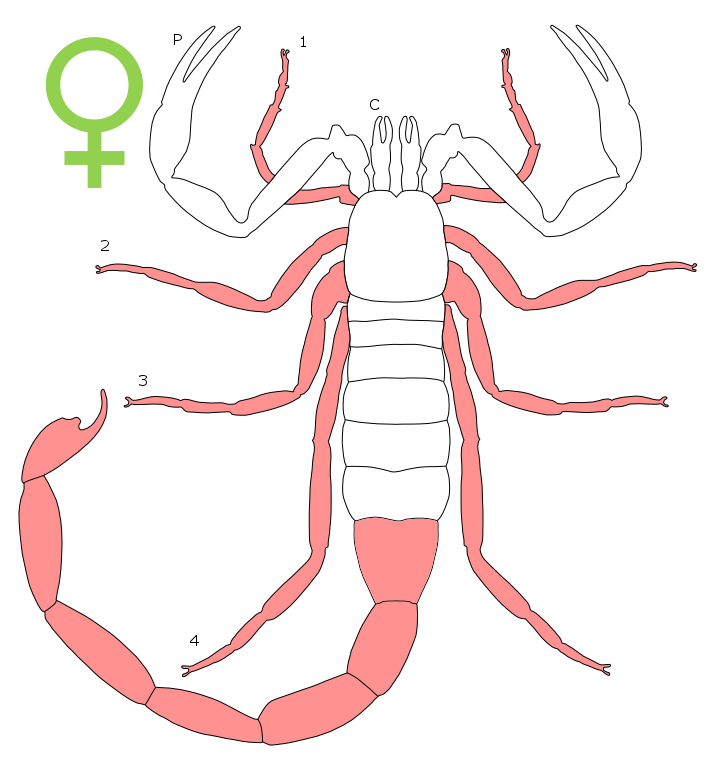

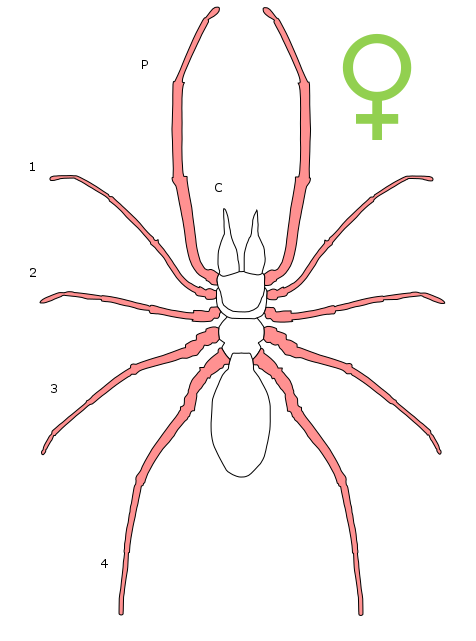

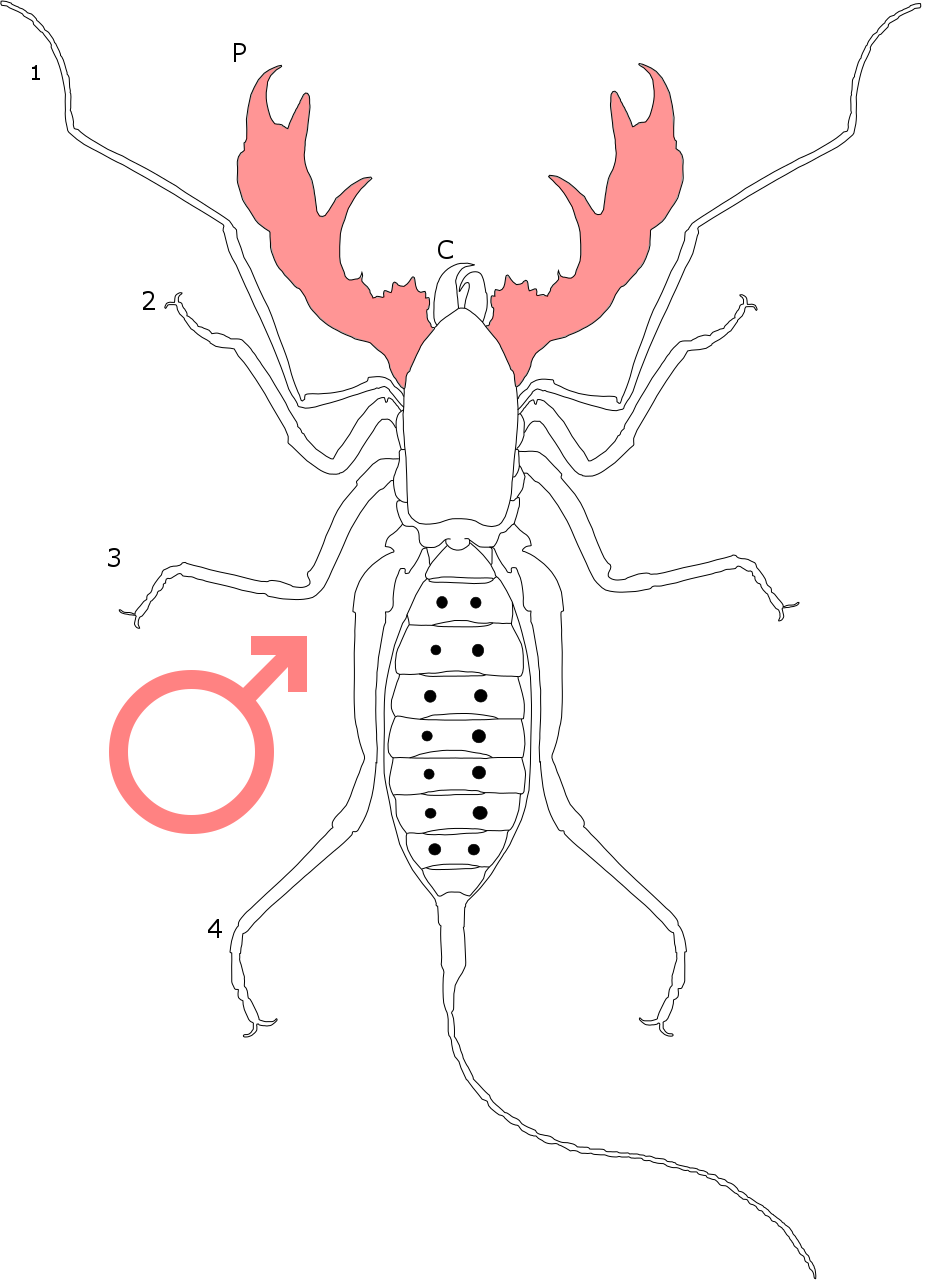

Supplement: Supplemental Information 2 — See “Standard Figure Abbreviations” for labelling guide. [file peerj-06-5751-s002.docx]
